# Long-Term Assessment of Surface Water Quality in a Highly Managed Estuary Basin

**DOI:** 10.3390/ijerph18179417

**Published:** 2021-09-06

**Authors:** Angelica M. Moncada, Assefa M. Melesse, Jagath Vithanage, René M. Price

**Affiliations:** 1 Department of Earth and Environment, Florida International University, Miami, FL 33199, USA; amonc002@fiu.edu (A.M.M.); pricer@fiu.edu (R.M.P.); 2 Institute of Environment, Florida International University, Miami, FL 33199, USA; 3 Sea Level Solutions Center, Florida International University, Miami, FL 33199, USA; jvithana@fiu.edu

**Keywords:** nutrients, statistical analysis, St. Lucie Estuary Basin, spatiotemporal trend, water quality, water pollution, dimensionality reduction

## Abstract

Anthropogenic developments in coastal watersheds cause significant ecological changes to estuaries. Since estuaries respond to inputs on relatively long time scales, robust analyses of long-term data should be employed to account for seasonality, internal cycling, and climatological cycles. This study characterizes the water quality of a highly managed coastal basin, the St. Lucie Estuary Basin, FL, USA, from 1999 to 2019 to detect spatiotemporal differences in the estuary’s water quality and its tributaries. The estuary is artificially connected to Lake Okeechobee, so it receives fresh water from an external basin. Monthly water samples collected from November 1999 to October 2019 were assessed using principal component analysis, correlation analysis, and the Seasonal Kendall trend test. Nitrogen, phosphorus, color, total suspended solids, and turbidity concentrations varied seasonally and spatially. Inflows from Lake Okeechobee were characterized by high turbidity, while higher phosphorus concentrations characterized inflows from tributaries within the basin. Differences among tributaries within the basin may be attributed to flow regimes (e.g., significant releases vs. steady flow) and land use (e.g., pasture vs. row crops). Decreasing trends for orthophosphate, total phosphorus, and color and increasing trends for dissolved oxygen were found over the long term. Decreases in nutrient concentrations over time could be due to local mitigation efforts. Understanding the differences in water quality between the tributaries of the St. Lucie Estuary is essential for the overall water quality management of the estuary.

## 1. Introduction

The water quality of coastal areas is vital for maintaining ecosystem functions and services on which our society relies [1,2]. Yet, our use and development of coastal watersheds continue to cause significant ecological changes [3,4,5]. Losses of natural habitats due to increases in urban populations, drainage canals, and agricultural activities have impacted the quality, quantity, timing, and distribution of freshwater inputs to estuaries [6,7,8]. Furthermore, modified water inputs to estuaries have triggered the loss of seagrasses, hypoxia, fish kills, and algal blooms [9,10].

Resource management agencies continue to develop restoration and management plans to improve the quantity and quality of freshwater inputs to coastal zones to mitigate anthropogenic impacts. Assessments of the effectiveness of these plans and continuous monitoring are necessary [11,12]. The robust datasets produced by continuous monitoring contain vital information for identifying water quality variability and improving local management plans. The use of multivariate statistical analyses on local continuous sampling data has proven effective in characterizing the water quality of coastal systems [13,14,15,16]. Since water quality is influenced by geographic region and local anthropogenic activities [17], the robust analysis of regional data can detect unique characteristics to be considered in tailored management plans [18,19]. 

Multi-year water quality data from water bodies across the US and elsewhere have been valuable for a better understanding of regional systems [20,21,22,23]. Multivariate statistical analyses and nonparametric tests are effective at summarizing robust datasets. For example, Stets et al. (2015) [20] used correlation analysis and principal component analysis to analyze 63 years of nitrate data from 22 sites across the US. They found nitrate was strongly related to agriculture, and it was higher in the mid-west and less so in the east and west. Similarly, Boyer et al. (1999) [21] characterized the water quality of three distinct zones of Florida Bay using a 6-year dataset and found that turbidity increased by a factor of 20 at one of the bay areas.

Furthermore, a 20-year water quality dataset from various Texas Gulf coastal sites analyzed by Bugica et al. (2020) [22] revealed two waterbodies showed signs of eutrophication throughout the study period even though river inputs did not influence them. They concluded that non-point and point-source loads and residence time were the main factors driving eutrophication at those sites. Romero et al. (2016) [23] used a 40-year water quality dataset of the lower Seine River, France, and found a turning point in the 1990s when decreases in ammonium and phosphate were due to the ban of phosphates and wastewater treatment, whereas nitrate inputs increased due to agricultural practices. These findings exemplify the effectiveness of applying statistical analyses to multi-year datasets, especially in those areas showing water quality deterioration.

The St. Lucie Estuary, in east-central Florida, is one of many coastal areas displaying undesirable ecological shifts due to anthropogenic eutrophication and modified freshwater regimes [24,25]. Currently, the St. Lucie Estuary is a phytoplankton-based system that no longer supports permanent or extensive populations of oysters and seagrass [26,27]. The St. Lucie Estuary receives freshwater inputs from canals, precipitation, and groundwater, but 70% of its freshwater inputs are from drainage canals [28], including an artificial connection to Lake Okeechobee. Thus, characterizing the water quality of the canals that discharge into the St. Lucie Estuary is essential for management purposes. 

Ecological, biochemical, and flow regime studies of the St. Lucie Estuary Basin have improved the knowledge base of best management practices [25,29,30,31] and eutrophication science [32,33,34,35]. Studies by Doering (1996) [29] and Chamberlain and Hayward (1996) [26] recommended a more stable, lower flow from canals to improve water quality and attain resource management goals. Qian et al. (2007) [30] assessed long-term data (1979 to 2004) of water quality constituents in major canals and found that almost all nutrient species had significantly higher concentrations in the wet than in the dry season. Studies by Hampel et al. (2020) [32], Kramer et al. (2018) [33], and Oehrle et al. (2017) [35] demonstrated the key role of nitrogen species and salinity in toxic *Microcystis* blooms in the lake–estuary continuum. The South Florida Water Management District (SFWMD) and the Florida Department of Environmental Protection (FDEP) continuously assess the water quality of the estuary and the lake. The latest publicly available document by the FDEP, the updated 2020 St. Lucie River and Estuary Basin Management Action Plan [25], assessed nutrient loadings after the implementation of total maximum daily loads (TMDL) in 2013 [36] and summarized the current standing of management plans. 

The water quality of this basin has been continuously monitored since the 1970s by the SFWMD, which has generated a robust historical dataset from which long-term patterns can be extracted. While these studies have established essential characteristics of the St. Lucie Estuary Basin using various statistical techniques, there are no published statistical approaches that reduce the dimensionality of the data to distinguish the main physicochemical variables characterizing the water quality of the tributaries and the estuary. Long-term relationships among the physicochemical variables from 1999 to 2019 of water quality have not been explored either. The general objective of this study was to characterize the water quality of the St. Lucie Estuary Basin, with particular attention to the surface tributaries from 1999 to 2019. Three specific objectives were framed: (1) characterize the seasonality of flow values of the major surface tributaries; (2) identify the principal water quality constituents; and (3) evaluate monotonic trends of all physicochemical variables for 20 years. Multivariate statistical approaches, nonparametric tests, and trends were used in this study.

## 2. Materials and Methods

### 2.1. Study Setting

**Figure 1:**
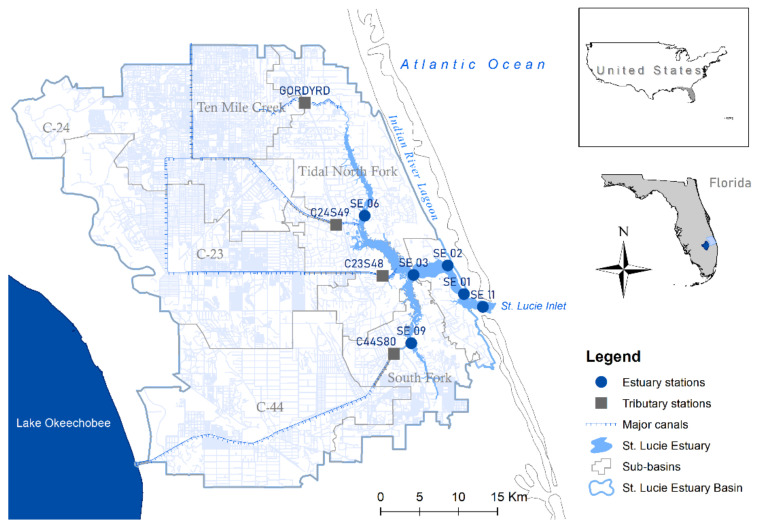
The St. Lucie Estuary Basin in the eastern Florida Peninsula, USA, the eleven water monitoring stations for water quality, the sub-basins, and the major canals draining to the estuary.

The St. Lucie Estuary is in the southern tip of the Indian River Lagoon, on the eastern coast of the Florida Peninsula, USA (Figure 1). The 28 km^2^ estuary is in the tidewater area at the junction of the North and South Forks of St. Lucie River and the Indian River Lagoon. The two forks converge along US-1, and the estuary extends another 9.7 km downstream to the Indian River Lagoon, which is connected to the Atlantic Ocean through the St. Lucie Inlet. A humid, subtropical climate prevails in this area, which is characterized by warm, wet summers (May to October) and mild, relatively dry winters (November to April) [29]. The annual atmospheric temperature ranges between 19 °C and 29 °C. The long-term total rainfall to the basin is 1234 mm per year, with 78% in the wet season and 22% in the dry season [37].

#### 2.1.1. Estuary

**Figure 2:**
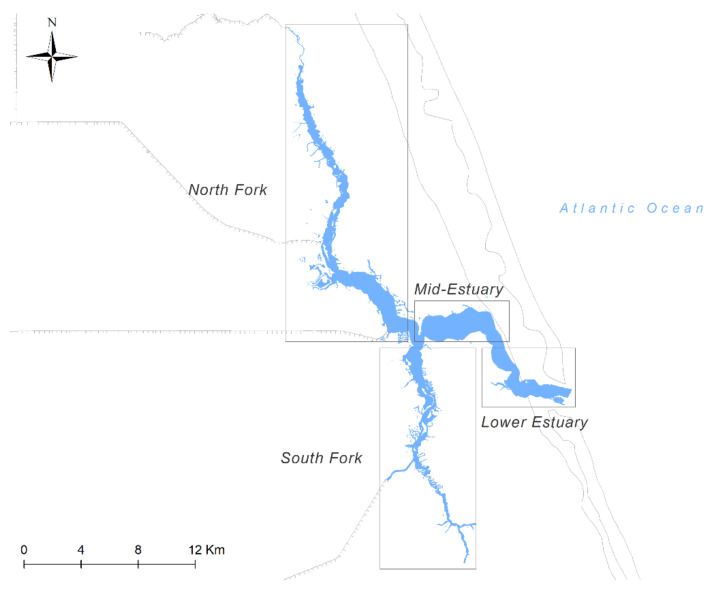
The four geographical sections of the St. Lucie Estuary, Florida, USA.

The St. Lucie Estuary has four main geographical sections: the North Fork, the South Fork, the Mid-Estuary, and the Lower Estuary (Figure 2), where salinity has intra-annual fluctuations. Salinity patterns affect productivity, population distribution, community composition, and food web structure in the estuary. In shallow and highly managed estuaries, such as the St. Lucie Estuary, salinity is driven mainly by hydrologic events and water management practices and can range from <1 to >35 parts per thousand [38,39]. Vertical stratification generally happens during large water releases from Lake Okeechobee. The Lower Estuary, the area closest to the ocean inlet, generally has the highest median salinity and the lowest salinity values are in the North and South Forks, which are furthest from the ocean inlet [40].

#### 2.1.2. Land Cover

The St. Lucie Estuary receives approximately 70% of its freshwater from four surface tributaries [41]: the Ten Mile Creek, Canal-24 (C-24), Canal-23 (C-23), and Canal-44 (C-44) (Figure 1). Water inputs from the Ten Mile Creek, C-24, and C-23 are solely from within the estuary’s drainage basin [42], while those from C-44 include periodic releases from Lake Okeechobee. The major land cover classes for the St. Lucie Estuary Basin are cultivated crops, hay/pasture, and wetlands [43]. The Ten Mile Creek sub-basin is 158 km^2^, and its major land-cover classes are cultivated crops (50%), hay/pasture cover (34%), developed-open space (6%), woody wetlands (5%), and developed-low intensity (2%). Sub-basin C-23 is 448 km^2^, and its major land cover classes are hay/pasture (38%), cultivated crops (24%), woody wetlands (23%), emergent herbaceous wetlands (4%), and developed open space (3%). Similar to C-23, sub-basin C-24 is 425 km^2^, and its major land cover classes are hay/pasture (38%), woody wetlands (26%), cultivated crops (17%), developed open space (4%), and developed low intensity (3%) [43]. C-44 combines basin runoff from sub-basin C-44 with Lake Okeechobee releases. This artificial connection with Lake Okeechobee brings in water from a 14,000 km^2^ area where 36% are improved pastures, 21% wetlands/water bodies, 16% rangeland/unimproved, pasture, 10% forested uplands, 5% citrus, and 3% urban.

### 2.2. Data

#### 2.2.1. Rainfall and Flow

Rainfall (mm day^−1^) and canal flow (m^3^/s) for the 20 years of November 1999 to October 2019 were queried from the SFWMD’s publicly available repositories. Rainfall was obtained from the NEXRAD repository [44] using the polygon selection tool of the same extent as the estuary basin. The flow was gathered from the hydrogeologic database DBHYDRO [45] for stations named by the SFWMD as GORDYRD, C23S48, C24S49, and C44S80 (Figure 1).

#### 2.2.2. Water Quality Data

Physicochemical data for 20 years (November 1999–October 2019) from ten water monitoring stations were also obtained from the SFWMD’s publicly available repository DBHYDRO [46]. The monitoring stations were selected based on data continuity over the 20 years and their distribution throughout the basin. Stations named C23S48, C24S49, GORDYRD, and C44S80 (Figure 1) by the SFWMD represent the freshwater tributaries C-23, C-24, Ten Mile Creek, and C-44, respectively. Stations C23S48, C24S49, and GORDYRD are on canals that drain from within the basin of the estuary, while station C44S80 is downstream of Lake Okeechobee. Stations named by the SFWMD as SE 01, SE 02, SE 03, SE 06, SE 09, and SE 11 are in different regions of the St. Lucie Estuary (Figure 1). Station SE 06 is in the North Fork, SE 09 is in the South Fork, SE 02 and SE 03 are in the Mid-Estuary, and SE 01 and SE 11 are in the Lower Estuary (Table 1).

Values for eleven physicochemical variables were used: ammonia (NH_3_), color, dissolved oxygen (DO), nitrate + nitrite (N+N), pH, orthophosphate (OP), total phosphorus (TP), specific conductivity, total nitrogen (TN), total suspended solids (TSS), turbidity, and surface water temperature (SWT). Water samples were collected monthly by the SFWMD and analyzed in their analytical laboratory. The SFWMD followed either the Standard Methods [47], the United States Environmental Protection Agency (1987, 1979), or the SFWMD’s field sampling quality manual (SFWMD-FSQM) depending on the measured variable (Table 2). The samples from canals were collected at 0.5 m below the water surface, and those at the estuary were from the middle of the total depth of the water column at the time of collection. The mean depth of the estuary is 2.4 m [28].

TN values from November 1999 to September 2014 were unavailable from the SFWMD’s repository, but total Kjeldahl Nitrogen (TKN) and N+N were. Therefore, for that period, TN was calculated by the authors by adding the TKN and N+N values available from the repository. This procedure was consistent with that followed by the SFWMD [48]. TN values from 2014 to 2019 were downloaded from the repository. For the most part, SFWMD collected one sample a month from each station from November 1999 to October 2019.

The SFWMD staff uses the Data Collection/Validation Preprocessing (DCVP) system to perform quality assurance/quality control (QA/QC) on instrument readings before archiving the data. Preliminary time-series data are extracted from the DCVP and subjected to an initial QA/QC check to ascertain or improve data quality through the Graphical Verification Analysis (GVA) Program. The GVA application is used for the validation of the data. Data are uploaded into the DBHYDRO database after the GVA analysis [49].

The sample sizes of the physicochemical variables differed among stations. Supplemental Table S1 details the sample size for each variable at each station. There were some prolonged gaps in collection for certain stations, which spanned several months. These gaps caused some stations to have smaller sample sizes. The largest gap in the collection was at station SE 06 from July 2012 to March 2015. As a result, the sample size for NH_3_ was 173 versus a sample size of 242 at GORDYRD for the same variable. 

Some datasets had values below the detection limit (BDL), also called nondetects (Table S1). The substitution of values BDL was based on the percent contained in each dataset. Simple substitution to half the minimum detection limit value was used where nondetects comprised 10% or less of the dataset. The values used for simple substitution are specified in Table 2. For datasets containing over 10% of nondetects, robust regression on order statistics (ROS) was used. The robust ROS uses the sample data that are not BDL to assume the distribution and to assign values to those non-detects as described and suggested by Helsel and Cohn (1988) [50] and Helsel et al. (2012) [51]. 

### 2.3. Statistical Methods

#### 2.3.1. Assessment of Rainfall and Flow Data 

All statistical analyses were done using R Version 3.5.2. Descriptive statistics of rainfall and flow were calculated for the 20 years, and the intra-annual variability of flow for each canal was assessed visually using time-series charts. The correlation between canal flow and rainfall was assessed with Kendall’s tau correlation coefficient (τ) [52]. Kendall’s tau correlation coefficient indicated the strength of monotonic correlations (linear and nonlinear) between rainfall and flow. Tau is commonly used in water quality data analyses because it is resistant to data skewness and outliers [53,54,55]. Kendall’s tau (τ) correlation coefficient is a rank-based procedure where the test statistic (S) is calculated by subtracting discordant pairs from the number of concordant pairs. S is then divided by the number of possible comparisons to be made among the n data pairs as detailed in Equation (1): Kendall’s tau correlation coefficient

τ = S/(n(n − 1)/2)(1)

where a τ value close to −1 or 1 indicates strong negative or strong positive monotonic relationships between the variables, respectively, and τ values close to or equal to 0 indicate no relationship between them. However, due to its ranking nature, τ coefficients are smaller than those of the more commonly applied linear correlation coefficients such as Pearson’s R. A strong linear correlation of 0.9 or above corresponds to τ values of about 0.7 or above [56]. In this study, when τ is between 0.7 and 1, it is considered a strong correlation; between 0.4 and 0.7 a moderate correlation; and between 0.2 and 0.4 a weak correlation.

#### 2.3.2. Assessment of Physicochemical Variables

Histograms were used to explore the distribution of the values of each physicochemical variable at each station when selecting the appropriate statistical analyses. The summary statistics for the physicochemical variables were reported separately for the wet (May–Oct) and dry (Nov–Apr) seasons for each station (Table S2). The arithmetic mean, the standard deviation, the median, and the interquartile range were reported for each water quality variable for the 20 years. The mean and interquartile range (IQR) for pH were determined as the negative logarithm of the average hydrogen ion concentration.

#### 2.3.3. Principal Component Analysis (PCA)

PCA has proven to reduce the redundancy of large multivariate water quality datasets for identifying and summarizing patterns [39,57]. By transforming the original variables into new uncorrelated variables, called principal components or dimensions, the noise is reduced while preserving as much of the data’s variation as possible [58]. Each monthly water sample was a variable in this study, and each physicochemical variable was a factor. PCAs were done separately for tributary samples and estuary samples to identify the principal variables that characterize the water in these different areas. The physicochemical variables used in the PCA were NH_3_, N+N, TN, OP, TP, color, TSS, turbidity, DO, and pH.

#### 2.3.4. Correlation and Trend Analyses

The correlation and trend tests performed were selected based on the nonparametric nature of the datasets. The strength of monotonic correlations between the physicochemical variables and flow was measured using Kendall’s tau correlation coefficient (τ). The R script for the pair plots was modified from Ryberg (2017) [59]. The Kendall’s tau correlation and significance were assessed for the physicochemical variables from tributary samples. Only tributary samples were chosen because they presented stronger associations in the PCA than samples from the estuary and greater applicability in water management as these inputs are important drivers of the estuary’s water quality. NH_3_, color, DO, pH, TP, specific conductivity, SWT, TN, turbidity, and flow were assessed for the 20 years. 

The monotonic trends of variables for the 20 years were assessed using the Seasonal Kendall trend test (*S_k_*) [60], which accounts for seasonality by calculating the Mann–Kendall [61] test for each month separately and then combining the results. The Mann–Kendall follows the same principle as Kendall’s tau but with time as the *x*-variable. The Mann–Kendall determines whether the central value (median) changes over time. The S statistic, *S_i_*, for each month are summed to form the overall statistic *S_k_* as in Equation (2):

(2) $$S_{k}=\overset{m}{\underset{i=1}{\sum }}S_{i}$$

The equation was applied to data collected monthly. For example, all data obtained in May were compared to similar data collected in May throughout the 20-year study period. The process was repeated for data collected in June and so on for all the months. Since this method works best with no missing data, missing values were filled in using a predictive mean matching (PMM) imputation approach. This imputation method fills the values using a simulated regression model [62]. 

The magnitude of the trend was evaluated by calculating Sen’s slope [63] for those variables showing significant *S_k_*. The nonparametric Sen’s method estimates the slope for the sets of pairs (*i*, *x_i_*), where *x_i_* is a time series. The Sen slope is calculated by:

(3) $$\beta =\mathrm{Median}\left(\frac{x_{j}-x_{i}}{j-i}\right),\;j>i$$

where *x_j_* and *x_i_* are the data values at times *j* and *i*, respectively.

The *β* is the median of all the slopes calculated for the selected time series. For this study, the period for each slope was one year. The Sen’s slope represents the median change per year for the 20-year study period. *β* > 0 indicates an upward trend. 

## 3. Results

### 3.1. Assessment of Rainfall and Flow Data

The summary statistics (Table 3) showed higher rainfall values from May to October, with August having the highest mean of 194 mm and median of 179 mm. Lower rainfall values were from November to April, with February having the lowest mean rainfall of 43.6 mm and median of 42.2 mm. Mean monthly flow values for June to October at C-23, C-24, and Ten Mile Creek were higher than the remaining months. In the wettest months, flow values at C-23 and C-24 were two to five times greater than their dry-month flow values. However, flow at Ten Mile Creek did not display such intra-annual differences. The Ten Mile Creek station displayed a steady flow with few peaks and zero no-flow conditions. At C-44, the mean monthly flow was highest from July to August, with some monthly values two- or three-fold higher than those of the other tributaries. C-44 had long periods of no-flow combined with abrupt spikes of very high flow (150 m^3^/s) compared to the other tributaries. Moreover, most of the observed peaks at C-44 coincided with the end of the wet season (October) and reflected large sporadic releases from Lake Okeechobee. 

Rainfall and flow were strongly correlated at tributaries C-23 and C-24 and weakly correlated at the Ten Mile Creek. Rainfall and flow had a τ of 0.53 for C-24, 0.51 for C-23, and 0.37 for Ten Mile Creek at α = 0.001. C-44 flow had a negligible τ of 0.15, α = 0.01. 

### 3.2. Assessment of Physicochemical Variables

The values of the physicochemical variables conformed to various distribution types, but for most stations, nutrient species, TSS, and turbidity values conformed to log-normal and exponential distributions while DO and pH values were normally distributed. At tributary stations, specific conductivity values were right-skewed for some stations and normally distributed for others while left-skewed at all estuary stations. The summary statistics for physicochemical variables were reported based on the intra-annual analysis of rainfall by separately evaluating the wet and dry seasons’ values (Supplementary Table S2). 

Nutrient concentrations were higher at the tributaries than at the estuary, and values were higher in the wet season, except for N+N, which was higher in the dry season for most stations. Nutrient species concentrations differed among the tributaries. Basin tributaries (C-23, C-24, and Ten Mile Creek) displayed higher nutrient concentrations than C-44 (influenced by Lake Okeechobee releases) except for N+N, which was highest at C-44. C-24 and C-23 had the highest concentrations of NH_3_ and TN of all stations; the mean value for NH_3_ was 0.1 mg/L at both stations, and the medians were 0.08 mg/L and 0.09 mg/L at C-24 and C-23, respectively. Similarly, the mean concentration of TN was 1.4 mg/L, and the median was 1.5 mg/L at both stations. C-23 and Ten Mile Creek had the highest OP and TP mean concentrations of 0.28 mg/L and 0.35 mg/L, respectively, but C-23 had the highest medians of 0.26 mg/L for OP, and 0.33 mg/L for TP C-44 had the highest N+N concentration with the same value of 0.3 mg/L for the mean and median. The lowest NH_3_ (0.03 mg/L), OP (0.062 mg/L), and TP (0.13 mg/L) mean concentrations were in the dry season at C-44. The lowest N+N (0.05 mg/L) mean concentration was at C-24 in the wet season. TN (0.81 mg/L) was lowest at Ten Mile Creek in the dry season. 

Nutrient concentrations in the estuary generally decreased with proximity to the ocean inlet as specific conductivity increased. Accordingly, the Lower Estuary had the lowest nutrient concentrations. NH_3_ was highest in the wet season at both the Mid-Estuary and South Fork with a mean concentration of 0.09 mg/L and median of 0.07 mg/L. N+N and TN were highest at the South Fork with a mean of 0.1 mg/L and median of 0.07 mg/L for N+N and a mean and median of 1.3 mg/L for TN; OP and TP were highest at the North Fork with a mean of 0.20 mg/L and median of 0.18 mg/L for OP and mean of 0.27 mg/L, and median of 0.26 mg/L for TP. 

Color values were highest at the tributaries and decreased with proximity to the ocean inlet. The highest color values were at C-23 and C-24 in the wet season, with a mean of 150 PCU and 149 PCU, respectively. TSS and turbidity were generally higher at the estuary than at the tributaries; however, tributary C-44 had substantially higher values than other areas. In the wet season, the mean values of TSS at C-44 were 13 mg/L and the median was 8 mg/L, while at the other tributaries, the mean values were between 3–5 mg/L and median values were between 1–4 mg/L. The highest mean turbidity value was also at C-44 (17 NTU); however, it was in the dry season while it was higher in the wet season in most stations. The mean turbidity at the other tributaries ranged between 2.8 and 4.4 NTU, with median values between 2.3 and 3.8 NTU. In the estuary, color and turbidity values were highest at the South Fork, but the highest mean color values (103 PCU) were in the wet season, while the highest turbidity values (10 NTU) were in the dry season. The lowest color values were at the Lower Estuary, with a mean of 7.8 PCU. TSS values were lowest at Ten Mile Creek, with a mean value of 3 mg/L and a median value of 1 mg/L. 

DO, pH, and specific conductivity values were generally higher in the dry season and at the estuary compared to the tributaries. The DO and pH values had lower variances at the estuary than at the tributaries. The highest DO and pH mean values were at C-23 and the Lower Estuary, with a mean of 7.0 mg/L for DO and 7.9 for pH in the dry season. DO and pH were lowest at Ten Mile Creek, with a mean of 3 mg/L for DO and 7.1 for pH in the wet season. Specific conductivity was highest at the Lower Estuary in the dry season with a mean value of 49 mS/cm and median of 51 mS/cm, and lowest at C-44 in the wet season with a mean of 0.66 mS/cm and median of 0.56 mS/cm. The SWT did not vary significantly across the tributaries nor the estuary, ranging between 21.1 and 22.4 °C in the dry season and 28.0 and 29 °C in the wet season. 

### 3.3. Principal Component Analysis (PCA)

**Figure 3:**
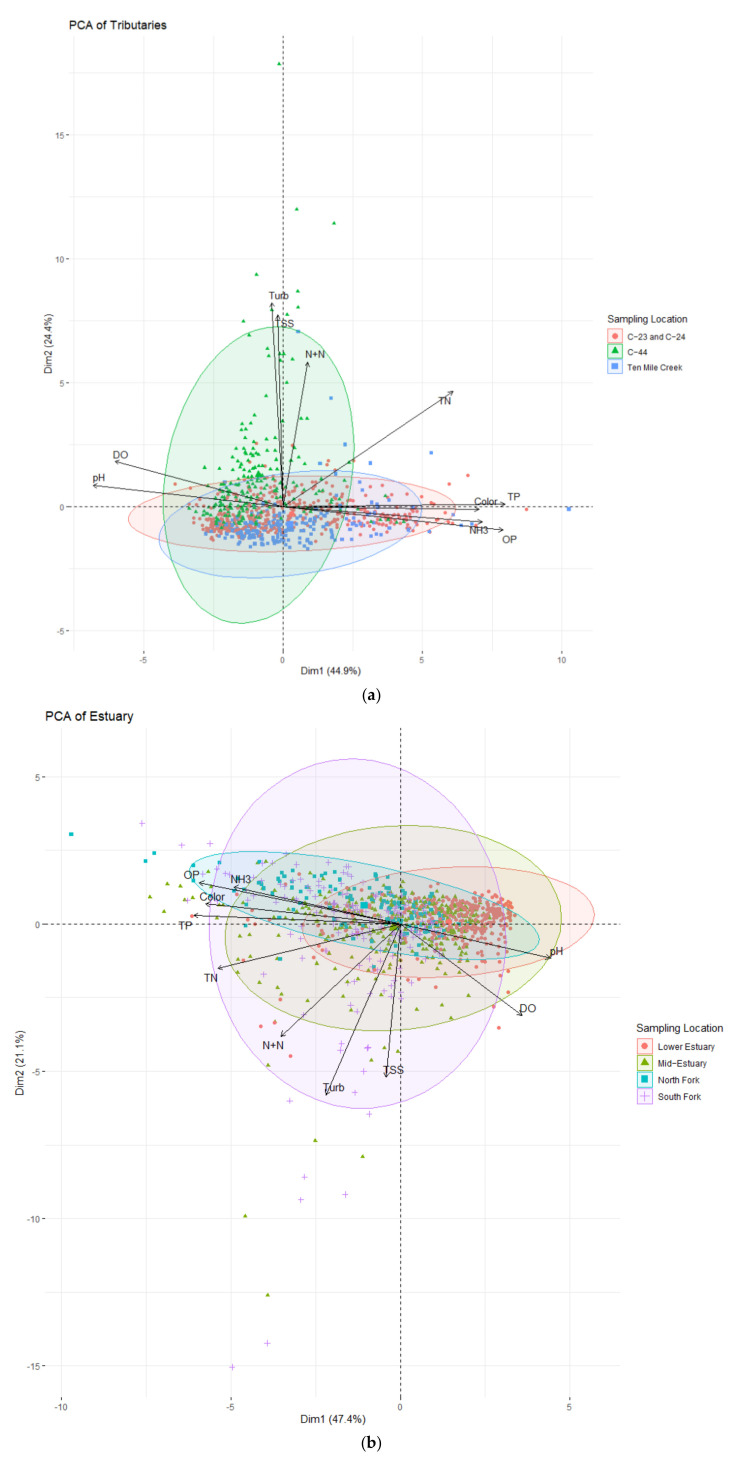
(**a**). Biplot of PCA of samples taken from the four main surface tributaries. (**b**). Biplot of PCA of samples taken from the St. Lucie Estuary.

**Figure 4:**
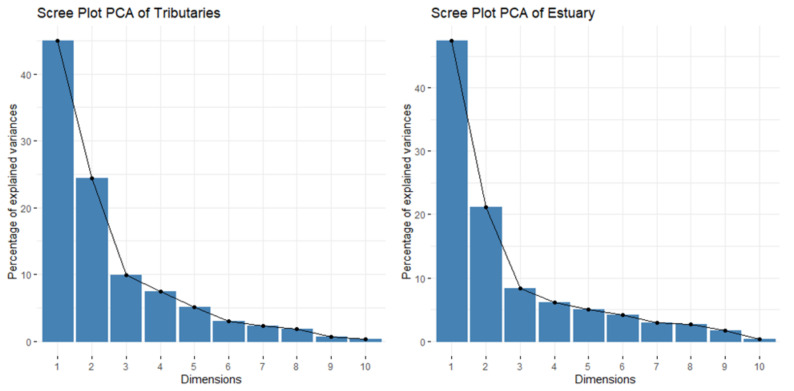
The scree plots of the PCA of samples from the tributaries (**left**) and those from the estuary (**right**) indicate the percent variance explained by each dimension.

The PCA biplots (Figure 3a,b) display the transformed data on the first two dimensions. The scree plots showed the percentage of variance accounted for by each dimension (Figure 4) and the loadings to each of the dimensions are shown in Table 4. The variables with more weight on the dimension were depicted with longer arrows than those with less weight on the biplots. Small angles between vectors represented a positive correlation, while those close to 180° were negatively correlated. The scree plots (Figure 4) showed that the first three dimensions of the tributaries accounted for 79.3% of the total variance, and the first three dimensions of the estuary accounted for 76.8%. However, for general characterization purposes, the first two dimensions were examined in more detail because they explained close to 70% of the variance, and the eigenvalue of dimension 3 was lower than 1 for both the tributaries and estuary samples.

The PCA biplot of the tributaries (Figure 3a) showed a pronounced difference between basin runoff (C-23, C-24, and Ten Mile Creek) and Lake Okeechobee inputs (C-44). Overall, Dimension 1 (Dim1) explained 44.9% of the variance (Figure 4 left). The highest positive loads for Dim1 of the tributaries (Table 4) were TP (0.43), OP (0.42), color (0.38), and NH_3_ (0.38), and the greatest negative loads were pH (−0.37) and DO (−0.32). Dimension 2 (Dim2) explained 24.4% of the variance among tributary samples (Table 3) with turbidity (0.60), TSS (0.56), and N+N (0.42) contributing the largest loads (Table 4). Samples from tributaries draining the basin were spread throughout the Dim1 axis but stayed close to zero on the Dim2 axis (Figure 3a). This distribution indicated a large variance in phosphorus, color, and NH_3_ values and a relatively small variance in turbidity and TSS. Inversely, the samples from C-44, influenced by lake releases, varied more across the Dim2 axis while tightly grouping in quadrant II. The spread across axis Dim2 of the C-44 samples indicated a large variance in turbidity, TSS, and N+N and comparatively low variance and values for TP, PO, color, and NH_3_.

The biplot of the estuary (Figure 3b) showed the North Fork (SE 06) to be influenced by high values and variance in phosphorus, color, TN, and NH_3_, and low variance in turbidity and TSS values. The Mid-Estuary (SE 03 and SE 02) also showed more variance in Dim1 than Dim2; however, notable outliers were driven by turbidity and TSS. The Lower Estuary samples are most tightly grouped to the right of Dim1 origin and have low variance in both dimensions. Samples from the Lower Estuary were tightly grouped on Dim1, where pH and DO have the greatest loading. The South Fork (SE 09), downstream of lake inflows, showed high variances for all variables. However, samples mainly were distributed to the left of Dim1′s origin, indicating a higher influence from phosphorus and its related variables than pH and DO. There were notable outliers driven by phosphorus and its associated variables and outliers driven by turbidity and TSS. For the PCA of the estuary, Dim1 explained 47.1% (Figure 3b) of the variance with TP (−0.42), OP (−0.41), color (−0.40), and TN (−0.37) the greatest negative loads and pH (0.31) and DO (0.25) the only positive loads. Dim2 explained 21.1% of the variance, with turbidity (−0.60) and TSS (−0.54) contributing the highest loadings (Figure 4 and Table 4). 

An inverse relationship between phosphorus and the variables pH and DO was observed on the PCAs of the tributaries and the estuary. The relationships between those dimensions characterizing the estuary were like those of the tributaries, with the difference that the correlations were more pronounced in the tributary dataset. 

### 3.4. Correlation of Physicochemical Variables

**Figure 5:**
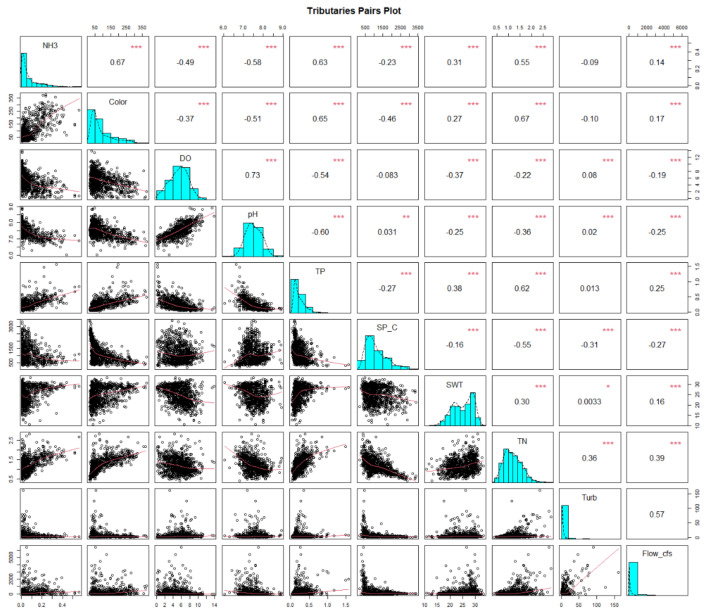
Pairs plot displaying Kendall’s tau correlation coefficient and *p*-value between water quality variables from 1999 to 2019. Central diagonal: variable names and histograms with a density line. Upper right half: Kendall’s tau correlation coefficient and *p*-value. Lower left half: scatter plots with loess smoothing. For *** *p* < 0.001, ** *p* < 0.01, * *p* < 0.05.

Kendall’s tau coefficient’s strongest significant positive correlation was between DO and pH (0.73) with a linear relationship (Figure 5). The next greatest positive tau coefficients were between NH_3_ and color (0.67), TP (0.63), and TN (0.55). The relationships among these variables were nonlinear. Moderate positive relationships (τ > 0.55) were indicated among color, TP, and TN with nonlinear relationships. TP and TN had a weak positive association with SWT and a moderate negative relationship with pH and DO. Strong negative correlations were found between pH and TP (−0.60) and between pH and color (−0.51), both with nonlinear relationships. Specific conductivity was moderately negatively related with both color (−0.46) and TN (−0.55). The relationships among the other variables were weak (τ < 0.5).

### 3.5. Trend Analysis

The Seasonal Mann–Kendall and Sen’s slope tests were mostly insignificant except at Ten Mile Creek and SE 06, both located in the North Fork (Table 5). Significant trends were weak, with only one moderate coefficient observed for OP at Ten Mile Creek. Other areas of the estuary basin showed significant but weak trends for certain variables.

The Ten Mile Creek had moderate (−0.45) decreasing trends in OP values and weak decreasing trends in TP (−0.36) and color (−0.22) values. The estimated Sen’s slope for OP and TP at this station showed a monthly decrease of 0.008 mg/L per month. This tributary also had increasing trends in DO (0.19), pH (0.25), SWT (0.28), and turbidity (0.31) values. The physicochemical variables at SE 06 typically showed the same trends as the Ten Mile Creek except for some variables which did not have significant trends.

Weak decreasing trends of TN concentration were detected at all sections of the basin. The Lower Estuary had the strongest decreasing coefficients for TN (−0.21), and N+N (−0.25), with Sen’s slopes, indicated a decrease of 0.02 mg/L and 0.0006 mg/L per month, respectively. 

## 4. Discussion

### 4.1. Seasonality of Freshwater Inputs

Rainfall is one of the main factors influencing temporal variability in water quality in the St. Lucie Estuary. Rainy months increase runoff and leachates, coinciding with higher temperatures and lower salinity upstream [26,29,30,64]. Thus, the assessment of rainfall allowed for the differentiation of variable concentrations for the wet and dry seasons. Intra-annual rainfall values for the selected period are consistent with a study by Qian et al. in 2007 [30], where the wet season was defined from 27 May to 7 November and the dry season from 8 November to 26 May. However, between 1979 and 2004, June had the highest mean monthly rainfall (192 mm) and December the lowest (49 mm). Our study (1999–2019) found that August had the highest mean monthly rainfall (193.6 mm) and February had the lowest (43.6 mm). Our results suggest a shift of two months in the wettest and driest months since Qian et al. (2007) [30].

While these differences in monthly rainfall could be explained by different sample sizes or regular multidecadal oceanic and atmospheric patterns [65,66], they can also be explained by a shift in precipitation patterns observed in Florida. A late onset of the wet season has been reported for some areas in the state, with a decrease in mean precipitation in May [67,68]. Moreover, statistically significant increases in rainfall for June and August were reported for South Florida by Abiy et al. (2019) [69] from 1906 to 2016. Their study coincides with our observed highest rainfall in August and the lowest rainfall month (February) to be later in the water year than previously reported.

At the St. Lucie Estuary Basin, rainfall was highly correlated with the flow of tributaries C-23 and C-24 but weakly associated with the flow at Ten Mile Creek and C-44. The differences in flow among the tributaries could be due to varying physical structures and management schemes. Canals C-23 and C-24 were constructed mainly to remove excess water from the basin. These linear canals are responsive to rainfall events and efficient at discharging runoff [24,70]. The C-44 canal is structurally similar to C-23 and C-24; however, its functions include discharging water from Lake Okeechobee and providing a navigable waterway from the lake to the coast. Thus, the C-44 is larger with a greater flow and water-holding capacity. For C-44 to sustain a navigable waterway, it has months with no-flow values to maintain the stage level. Different from the previously discussed tributaries, Ten Mile Creek is a more natural watercourse, for the most part, with a riparian buffer and some channelized sections. The lack of channelization may explain the weaker correlation with rainfall and low monthly flow variance observed for the Ten Mile Creek since channelization generally increases flood peaks and diminishes low flow values [71] as observed with the canals.

### 4.2. Seasonality of Water Quality

Seasonality was evident in the values of the physicochemical variables and their correlations. Mean and median concentrations of NH_3_, TN, N+N, OP, TP, color, TSS, and turbidity were generally higher in the wet season, while mean and median values for DO, pH, specific conductivity, and SWT were higher in the dry season. Seasonality was also evident in the correlation analysis among these variables; NH_3_, TP, TN, and color values were positively correlated with each other and negatively correlated with DO and pH. Higher concentrations of color and nutrients in the wet season are consistent with the results of previous studies done in the surface water of the St. Lucie Estuary Basin. A multiple regression that included freshwater discharge, water quality constituents, and salinity in seasonal time scales (wet and dry seasons) by Doering (1996) [29] explained up to 93% of the variation in estuarine water quality, suggesting that transport processes and mixing with ocean were relatively less important on seasonal than on monthly time scales in the St. Lucie Estuary. 

Previous studies have proposed multiple factors affecting the water quality of the estuary to explain the higher concentrations of nutrients in the wet season. Higher rainfall can lead to increased infiltration through soils in the basin, thereby mobilizing nutrients from fertilizers placed in the soils and increasing the groundwater table to compromise shallow septic tank systems [72,73,74,75,76]. Li et al. (2016; 2017) [72,73] measured different phosphorus and nitrogen species across the basin and found them to be positively correlated with rainfall. They also found phosphorus concentration was highest after the first runoff events following the application of fertilizers. Millie et al. 2004 [74] found higher nutrient enrichment in the wet season at the North Fork and attributed it to the releases of runoff from drainage canals into the estuary during periods of high rainfall. In addition to surface water discharges, groundwater seepage from septic systems in the St. Lucie Estuary Basin is a significant nutrient loading source that varies seasonally [34,75]. Lapointe et al. (2017) [34] and Barile (2018) [75] attributed an increased mobilization of ammonia in the wet season to a decrease in retention time due to higher water tables. This decrease in retention time was also associated with higher N+N concentrations during the dry season at the tributaries. Higher retention rates in the dry season at the septic systems allow for nitrification and the conversion of ammonia to N+N. Additionally, Li et al. (2016) [72] associated higher nitrate leaching in the dry season with minimal plant uptake in agricultural areas and a slower nitrification rate due to higher soil moisture in the wet season. 

Increased inputs from Lake Okeechobee in the wet season and resuspension of sediment and non-point source runoff from agricultural fields and point sources from stormwater, wastewater, and aquaculture are also considered sources of nutrients to the St. Lucie Estuary [76,77]. Lapointe et al. (2012) [76] found higher turbidity, nitrate, and dissolved inorganic nitrogen values at the South Fork than at the North Fork and Mid-Estuary after large discharges from the lake in the wet season. Additionally, Buzzelli et al. (2013) [64] found that when flushing times of the St. Lucie Estuary were lower than ten days, which is especially common in the wet season with increased discharges, resulted in a spike in autotrophy and increased N_2_ fixation, which could further increase nitrogen concentrations.

DO, pH, specific conductivity, and SWT values were generally higher in the dry season at most sites and were negatively correlated with nutrients. Lower DO and pH values in the wet season could be attributed to higher SWT and higher dissolved organic matter content in the water column [78,79,80]. Warmer water has a lower capacity of maintaining dissolved oxygen than colder water, and a high content of dissolved organic matter may increase microbial activity and lower DO concentration. An elevated amount of humic acid, fulvic acid, and tannins, which are weak acids, from dissolved organic matter have been documented to lower the pH in mangrove areas such as the North and South Forks of the estuary [78,79,80]. The negative correlations between nutrient concentrations with pH and DO indicate that the nutrients are more labile under lower redox conditions.

### 4.3. Spatial Variability of Physicochemical Variables

The physicochemical variables differed among the sampling stations. Mean and median phosphorus concentrations, nitrogen, color, TSS, and turbidity were higher in the tributaries and decreased downstream, while DO, pH, and specific conductivity were higher in the estuary and were negatively correlated with nutrients. Differences in nutrient concentrations and DO between the tributaries and the lower estuary may be attributed to dilution, tidal mixing, internal nutrient cycling, and settlement of suspended solids, among other processes [81]. Specific conductivity and pH were greatest in the Lower Estuary. A pH value of 8 and specific conductivity between 30–50 mS/cm were consistent with values typically observed in ocean water [82].

Runoff from the basin tributaries (Ten Mile Creek, C-23, and C-24) had higher TP, OP, NH_3_, and TN, while lake inflows (C-44) had higher turbidity, TSS, and N+N. Phosphorus concentrations at basin tributaries and high turbidity at C-44 were the principal factors distinguishing basin and lake inflows by the PCA. Mean TP at the basin tributaries was double that of C-44 in the wet season, while turbidity at C-44 was two- to three-fold that of basin tributaries. 

Differences among the tributaries may reflect the land-cover types and the agricultural practices of their respective sub-basins [79,83,84]. Higher phosphorus from basin tributaries compared to lake inflows is in line with findings by Zheng et al. (2016) [85], where the mass ratio of TN:TP indicated that water from the St. Lucie Basin tributaries was relatively enriched with TP compared to that from Lake Okeechobee. Lapointe et al. 2012 [76] found phosphorus was higher at the North Fork than the South Fork during large discharges from the lake, attributing it to fertilizer applications from golf courses, citrus, and row crops adjacent to C-23 and C-24. The main spatial differences among the basin tributaries were higher OP and TP at Ten Mile Creek and C-23, and higher TN, NH_3_, and color at C-23 and C-24. Graves et al. (2004) [78] found row cropland contained significantly higher phosphorus than other land uses, which is in line with higher OP and TP at Ten Mile Creek, where cultivated crops make up the largest cover-type (50%), followed by hay/pasture (34%). C-23 and C-24 basins did not have such a dominating land-cover type, but their largest cover type was hay/pasture with 38%, followed by cultivated crops at 25%. A larger hay/pasture area could explain higher TN and NH_3_ concentrations at C-23 and C-24. Graves et al. (2004) [78] found runoff from hay/pasture contained twice the nitrogen of runoff from cropland and had higher leaching of humic and tannic acids related to color. Similarly, the highest N+N values observed at C-44 may be related to the back-pumping of agricultural runoff into the lake, where 36% are improved pastures and 16% are range land/unimproved pastures [77,86,87]. Yang et al. (2013) [83] also attributed variations in dissolved nitrogen to the application of fertilizers, tillage management, and crop types. 

Within the estuary, the North Fork had the lowest mean and median concentrations of DO, reflecting the values of its tributary, Ten Mile Creek, and the estuarine circulation. Ten Mile Creek had the lowest DO values of all sites, with a mean of 3 mg/L and a median of 2.6 mg/L in the wet season. The low DO in this tributary may be related to enhanced primary productivity due to high phosphorus concentrations or low wind-induced mixing. Wan et al. (2012) [84] estimated low DO conditions in the North Fork during large discharges from both the basin and the lake, attributing them to a complex circulation pattern where large inflows from the lake combined with the tide and push water into the North Fork, thereby affecting vertical mixing. 

Of all sites, C-44 had the highest turbidity and TSS values. High turbidity in inflows from Lake Okeechobee was documented by James et al. (2009) [88] and Wang et al. (2012) [87] and was explained as a result of the resuspension of sediments in the lake water column due to wind-driven waves. Highly turbid inflows from the lake have been deemed undesirable in this estuary because they decrease light penetration to the bottom and impact submerged aquatic vegetation and bottom dwellers [87], thereby affecting biogeochemical feedbacks at the sediment-water interface [64]. 

The North Fork had the highest TP and OP concentrations and the lowest DO and color values among the estuary sites. The high phosphorus concentrations at the North Fork may be due to its upstream tributary, Ten Mile Creek, and groundwater seepage from adjacent urban areas. Seepage of reactive phosphorus from septic systems that are either existing or removed is an important source of reactive phosphorus in the North Fork [34,89]. Lapointe et al. (2017) [34] found reactive phosphorus was significantly higher in residential sites than non-residential sites due to septic systems. Their study sites were located adjacent to the St. Lucie Estuary. Ye et al. (2017) [89] found that the proximity (length of flow path) of the septic systems to the estuary was determinant in the loading potential. The proximity and high phosphorus seepage from septic systems may play an important role in phosphorus concentrations in the North Fork. A final source of phosphorus in the St. Lucie estuary could be the resuspension of sediments from the bottom of the estuary, which have high concentrations of water-soluble phosphorus within the upper 1 m [90]. 

The concentrations of NH_3_, N+N, and TN were highest at the South Fork and the Mid-Estuary. These high concentrations coincide with high concentrations from tributaries draining upstream, but also with adjacent septic systems, previously mentioned, which are also significant sources of nitrogen in this area [34,41,89]. Lapointe et al. (2017) [34] observed nitrogen was also seeping predominantly from residential sites. Ye et al. (2017) [89] also found septic systems a significant source of nitrogen loading relative to other agricultural and natural lands. The spatial variance of different nitrogen forms in this estuary is important in mitigating harmful algal blooms. Nitrogen is the element most capable of promoting cyanobacterial blooms in the lake and the estuary. Levels of TN, microcystin, and toxic *Microcystis* strains are highly and significantly correlated across the lake and estuary gradient. In addition, the competition between cyanobacteria and nitrifiers for ammonium impacts the capacity for denitrification in the system [32,33].

The FDEP set Total Maximum Daily Loads (TMDL) as water quality targets for both TN (0.720 mg/L) and TP (0.081 mg/L) in the St. Lucie Estuary [36]. The mean and median concentrations for TN computed for this study for 1999 to 2019 exceeded the TMDL year-round at all tributaries and the North Fork (SE 09) and South Fork (SE 06) of the estuary. Mean and median TN concentrations were also exceeded the Mid-Estuary stations (SE 03, SE 02) and the Lower Estuary (SE 01) but only in the wet season. Mean values of TN at the most downstream Lower Estuary (SE 11) remained below the TMDL. The mean and median concentrations of TP also exceeded the TMDL year-round at all tributaries, the South Fork, the North Fork, and the Mid-Estuary. TP TMDL concentrations at the Lower Estuary (SE 01 and SE 11) were only exceeded in the wet season. Our observations of mean seasonal TP being consistently higher than the TMDL for all stations upstream of the Lower Estuary indicates that releasing phosphorus into the estuary is a concern year-round.

### 4.4. Monotonic Trends

The observed monotonic decreases of NH_3_^+^, N+N, N, OP, TP, and color at various sections of the St. Lucie Estuary, particularly in the North Fork basin, could be due to shifts in flow management and the implementation of the St. Lucie River and Estuary Basin Management Action Plan (BMAP) [91] by the FDEP. Best management practices for decreasing nutrient loading and changes in hydrology have been effective methods for improving water quality at the Chesapeake Bay [92] and the Kissimmee River [93,94]. The observed decreasing trends in TP at the North Fork in this study were also reported in the 5-Year Review of the St. Lucie BMAP [95]. Their analysis of the Seasonal Kendall test for 2008 to 2017 showed a tau of −0.255 (*p* < 0.05) for TP in station SE 06. However, the 5-Year Review did not find significant increasing or decreasing trends in the values of any of the variables at the tributaries. The difference between this study’s findings and that of the SFWMD may be due to sample sizes. The FDEP assessed trends for a 9-year dataset while this study analyzed 20 years. Future research should implement change point detection methods to identify if significant decreases or increases in values of the physicochemical variables happen at one point in time.

The monotonic increase of pH and DO values at Ten Mile Creek and North Fork could be due to an increase in tidal mixing with seawater which is characterized by higher pH and DO values. Another factor explaining higher pH and DO could be increased submerged aquatic vegetation or algae in the North Fork. While there are no recent studies on submerged aquatic vegetation that we could find at the North Fork, there have been reports of moderately dense beds from the 1940s to the 1960s and unconfirmed reports of dense growths upstream of the estuary [96]. Increases in photosynthetic activity in the water column could lead to increases in pH and DO due to the removal of carbon dioxide [97], and it would also coincide with the monotonic decreases in nutrients concentrations in this same area. 

## 5. Conclusions

Shifts in rainfall and flow have implications for water quality and should be considered in long-term water quality analyses. Spatial differences in the flow and water quality constituents were observed across the St. Lucie Estuary Basin. Seasonality was evident in canal flow and for most water quality constituents. Flow values of basin-only canals were correlated with rainfall but flow at canal C-44, which connects to Lake Okeechobee, was not. Most water quality constituents were higher in the wet season than the dry season, likely due to increased infiltration, groundwater levels, and runoff. There were distinct spatial differences in water quality constituents across the tributaries, which could be explained by land cover and the different management regimes of the tributaries. The principal variables driving the water quality at the basin tributaries were OP, TP, color, and NH_3_^+^, while those driving Lake Okeechobee’s discharges were TSS and turbidity. Mean concentrations of TSS and turbidity on C-44 were two- or three-fold of those from basin runoff canals. Canals draining the central basins had the highest nitrogen and color mean values, while Ten Mile Creek had the highest OP and TP values. Nutrients were negatively correlated with pH and DO, possibly due to the seasonality of water temperature, dissolved organic matter, and microbial activity. OP, TP, and TN had moderate decreasing trends at the Ten Mile Creek and the North Fork, while DO and pH had moderately increasing trends due to changes in estuarine circulation, restoration efforts, and increased submerged aquatic vegetation at these sites. 

Multivariate data analysis tools such as PCA and nonparametric tests for monthly water quality data are important tools that should be more broadly applied in robust datasets. Results from PCA and trend analyses provide water managers with more information for guiding management plans. However, monthly data and data with minimum detection limits have limitations and bring challenges for analysis as these can be misleading or special cases. Studying the data distributions and the correct interpretations of nonparametric tests is important.

## Figures and Tables

**Table 1:** Location of water monitoring stations.

Monitoring Stations	Location	Latitude (N)	Longitude (W)
C23S48	C-23	27.2019	80.2992
C24S49	C-24	27.2614	80.3593
C44S80	C-44	27.1116	80.2850
GORDYRD	Ten Mile Creek	27.4030	80.3990
SE 01	Lower Estuary	27.1803	80.1939
SE 02	Mid-Estuary	27.2137	80.2148
SE 03	Mid-Estuary	27.2028	80.2592
SE 06	North Fork Estuary	27.2717	80.3220
SE 09	South Fork Estuary	27.1237	80.2625
SE 11	Lower Estuary	27.1653	80.1694

**Table 2:** Water quality variables. Abbreviations are those used in this paper and not by the management agency. Test methods are the methods used by the chemistry laboratory to obtain the values for each variable.

Variables	Abbreviations	Reporting Units	Test Methods	Minimum Detection Limit
Ammonia	NH_3_	mg/L	SM * 4500-NH3 H	0.009 (1999–2007); 0.005 (2007–2019)
Color	Color	PCU **	SM 2120 C	1
Dissolved oxygen	DO	mg/L	SFWMD-FSQM	NA
Nitrate + nitrite	N+N	mg/L	SM 4500-NO3-F	0.004 (1999–2004); 0.006 (2004–2007); 0.005 (2007–2019)
pH, field	pH	NA	SFWMD-FSQM	NA
Orthophosphate	OP	mg/L	SM 4500-P F	0.004 (1999–2007); 0.002 (2007–2019)
Total phosphorus	TP	mg/L	SM 4500-P F	0.004 (1999–2007); 0.002 (2007–2019)
Specific conductivity	Specific conductivity	mS/cm	SFWMD-FSQM	NA
Total nitrogen	TN	mg/L	Total Kjeldahl nitrogen (EPA 351.2) + nitrate + nitrite (1999–2014); Modified SM 4500-NC (2014–2019)	0.05 (1999–2014); 0.02 (2014–2019)
Total suspended solids	TSS	mg/L	EPA 160.2 (1999–2007); SM 2540 D (2007–2019)	3
Surface water temperature	SWT	Celsius	SFWMD-FSQM	NA
Turbidity	Turbidity	NTU	SM 2130 B	0.1

* SM = standard method by EPA; ** PCU = platinum cobalt unit.

**Table 3:** The monthly mean ($\bar{x}$) standard deviation (SD), median (Med), and minimum (Min), and maximum (Max) for rainfall and canal flow values for the period of November 1999–October 2019.

		Wet Season						Dry Season					
		May	June	July	August	September	October	November	December	January	February	March	April
Rain (mm)	$\bar{x}$	109	175	165	194	178	95.3	48.3	46.7	47.7	43.6	63.7	61.5
	SD	83.8	55.4	46.6	79.2	87.6	79.7	38.1	33.4	56.4	27.0	49.4	28.7
	Med	83.6	160	152	179	157	77.0	30.1	39.4	29.1	42.2	50.7	53.7
	Min	25.0	112	82.9	91.8	87.9	9.50	7.10	5.50	3.00	3.70	9.80	0.0
	Max	407	324	246	421	416	268	147	140	231	97.2	180	117
Ten Mile Creek Flow (m^3^/s)	$\bar{x}$	3.6	5.4	5.9	7.1	7.8	6.3	4.6	4.3	4.5	4.1	3.2	3.4
	SD	3.6	3.4	3.1	5.8	7.3	4.4	3.7	3.7	4.1	3.2	2.8	2.8
	M	2.5	3.9	4.9	4.9	5.5	4.7	2.8	3.2	2.5	3.6	2.2	2.0
	Min	0.1	1.3	2.3	2.0	2.7	1.1	0.6	0.7	1.4	0.8	0.9	1.2
	Max	14	13	15	28	35	15	13	15	14	12	11	10
C-24 Flow (m^3^/s)	$\bar{x}$	2.1	6.3	8.7	11	14	8.3	3.6	2.0	1.6	1.6	1.3	0.8
	SD	4.5	6.2	7.4	8.5	13	9.3	6.5	2.9	3.9	2.7	2.9	1.5
	M	0.6	4.4	6.7	9.0	10	5.5	1.2	0.6	0.2	0.1	0.1	0.0
	Min	0.0	0.0	0.0	0.0	1.2	0.0	0.0	0.0	0.0	0.0	0.0	0.0
	Max	19	24	25	34	61	29	27	11	17	11	9.6	5.5
C-23 Flow (m^3^/s)	$\bar{x}$	1.5	5.6	6.5	9.8	13	7.9	3.4	1.3	1.4	1.4	1.3	0.7
	SD	3.8	6.6	6.6	7.0	14	9.3	7.4	1.6	3.2	2.3	2.7	1.1
	M	0.2	3.2	3.8	6.9	11	4.9	1.0	0.7	0.5	0.3	0.3	0.1
	Min	0.0	0.0	0.4	2.4	2.1	0.2	0.0	0.0	0.0	0.0	0.0	0.0
	Max	17	27	22	31	65	35	33	5.3	14	9.7	11	4.4
C-44 Flow (m^3^/s)	$\bar{x}$	10	12	17	19	26	24	18	9.0	4.7	9.1	7.5	5.9
	SD	15	18	32	32	27	39	33	17	8.1	25	13	10
	M	0.2	0.1	5.8	4.4	19	5.1	1.2	0.6	0.6	0.4	0.0	0.0
	Min	0.0	0.0	0.0	0.0	0.0	0.0	0.0	0.0	0.0	0.0	0.0	0.0
	Max	43	52	128	122	99	144	104	69	32	112	52	32

**Table 4:** Variable loadings to the first three dimensions.

Variables	Tributaries			Estuary		
	Dim1	Dim 2	Dim 3	Dim 1	Dim 2	Dim 3
NH_3_+	0.383	−0.046	−0.094	−0.340	0.132	0.208
N+N	0.047	0.424	−0.264	−0.245	−0.322	0.481
TN	0.327	0.338	−0.345	−0.373	−0.157	0.152
OP	0.423	−0.068	−0.022	−0.410	0.147	0.051
TP	0.427	0.008	0.009	−0.422	0.030	−0.034
DO	−0.324	0.132	−0.554	0.248	−0.322	0.463
Color	0.377	−0.009	−0.414	−0.398	0.071	0.063
TSS	−0.011	0.564	0.307	−0.029	−0.542	−0.543
Turbidity	−0.022	0.600	0.240	−0.153	−0.603	−0.131
pH	−0.367	0.068	−0.415	0.308	−0.119	0.410

**Table 5:** The Seasonal Mann–Kendall test and the Sen’s slope were performed for the twenty years for each variable in each station to test for the significance, direction, and magnitude of non-seasonal trends.

	South Fork		Tributaries		North Fork		Mid-Estuary		Lower Estuary	
	C-44	SE 09	C-23	C-24	Ten Mile Creek	SE 06	SE 03	SE 02	SE 01	SE 11
NH_3_ τ slope	NT	▵ 0.14 −5 × $10^{-4}$	NT	NT	NT	▼ * −0.27 −5 × $10^{-4}$	NT	NT	NT	NT
N+N τ Slope	NT	NT	NT	NT	▽ −0.10 −1 × $10^{-3}$	▼ −0.18 −2 × $10^{-4}$	NT	▿ −0.14 −5 × $10^{-4}$	▼ −0.22 −6 × $10^{-4}$	▼ −0.25 −6 × $10^{-4}$
TN τ Slope	▼ −0.16 −1 × $10^{-2}$	NT	NT	▿ −0.09 −6 × $10^{-3}$	▼ −0.19 −1 × $10^{-2}$	NT	▽ −0.14 −8 × $10^{-3}$	▽ −0.14 −9 × $10^{-3}$	▼ −0.16 −1 × $10^{-2}$	▼ −0.21 −2 × $10^{-2}$
OP τ Slope	▽ −0.14 1 × $10^{-3}$	NT	▿ −0.11 −2 × $10^{-3}$	▽ −0.15 −2 × $10^{-3}$	▼ −0.45 −8 × $10^{-3}$	▼ −0.30 −4 × $10^{-3}$	NT	NT	NT	NT
TP τ Slope	NT	NT	▿ −0.11 −2 × $10^{-3}$	▽ −0.15 −3 × $10^{-3}$	▼ −0.36 −8 × $10^{-3}$	▼ −0.27 −4 × $10^{-3}$	▽ −0.13 −1 × $10^{-3}$	NT	NT	▿ −0.11 −7 × $10^{-4}$
Color τ slope	NT	NT	NT	NT	▼ −0.22 −0.820	NT	▿ −0.10 −0.330	▿ −0.10 −0.390	▽ −0.14 −0.330	▼ −0.17 −0.090
DO τ Slope	NT	NT	NT	NT	▲ 0.19 0.073	△ 0.13 0.047	▵ 0.12 0.023	△ 0.13 0.027	▲ 0.19 0.030	NT
pH τ Slope	NT	▵ 0.11 4 × $10^{-3}$	NT	▵ 0.09 7 × $10^{-3}$	▲ 0.25 8 × $10^{-3}$	▲ 0.34 2 × $10^{-2}$	NT	NT	NT	NT
Temp. τ Slope	NT	▵ 0.11 0.04	NT	△ 0.13 0.03	▲ 0.28 0.08	▵ 0.12 0.05	NT	NT	NT	△ 0.15 0.05
Sp. Con τ slope	NT	NT	▲ 0.28 12.4	▿ −0.10 −7.0	▲ 0.25 30.5	NT	NT	NT	NT	NT
Turb. τ Slope	NT	NT	NT	NT	▲ 0.31 0.10	▵ 0.12 0.00	NT	NT	▼ −0.16 −0.08	▽ −0.14 −0.07
TSS τ Slope	NT	NT	NT	NT	NT	NT	NT	NT	NT	NT

* The Kendall’s tau statistic (τ) is the number on top, and Sen’s slope is the bottom one. Symbols ▲▼ indicate an increasing or decreasing trend, respectively, at significance *p* < 0.001. Symbols ▽△ represent *p* < 0.01, and ▵▿ represent *p* < 0.05. NT indicates no significant (*p* < 0.05) monotonic trend.

## Data Availability

Publicly available datasets were analyzed in this study. This data can be found here: (DBHYDRO; https://apps.sfwmd.gov/ nexrad2nrdmain.action, accessed on 15 May 2020) and (DBHYDRO; https://my.sfwmd.gov/dbhydroplsql/water_quality_interface.main_page, accessed on 15 May 2020).

## Supplementary Materials

The following are available online at https://www.mdpi.com/article/10.3390/ijerph18179417/s1, Table S1: Percentage of data below the detection limit (BDL) and not reported (NR) for each station and variable, and Table S2: Summary statistics for dry and wet seasons of all physicochemical variables analyzed for all stations from November 1999 to October 2019.
