# Supplementary material for: Long-Term Assessment of Surface Water Quality in a Highly Managed Estuary Basin
Source: Int J Environ Res Public Health. 2021 Sep 6;18(17):9417. doi: 10.3390/ijerph18179417 (PMC8431535; doi:10.3390/ijerph18179417)
Supplement: Supplementary file 1 [file ijerph-18-09417-s001.zip › ijerph-1351151-supplementary.pdf]

Table S1. Percentage of data below the detection limit (BDL) and number of samples (N) for each station and variable.

	GORDYRD		C24S49		C23S48		C44S80		SE 09		SE 06		SE 03		SE 02		SE 01		SE 11	
	% BDL	N	% BDL	N	% BDL	N	% BDL	N	% BDL	N	% BDL	N	% BDL	N	% BDL	N	% BDL	N	% BDL	N
**NH_3^+**	0	242	11	231	12	221	11	233	11	213	9	175	8	212	14	206	23	204	36	202
**Color**	0	252	0	241	0	232	0	245	0	223	0	193	0	225	1	223	13	222	26	222
**DO**	0	257	0	238	0	231	0	241	0	222	0	191	0	222	0	225	0	221	0	224
**N+N**	2	249	28	231	23	226	13	242	11	220	29	186	11	221	20	217	30	216	33	216
**pH**	0	259	0	240	0	232	0	246	0	225	0	194	0	224	0	224	0	224	0	224
**OP**	0	252	1	239	0	232	0	245	0	220	0	190	0	222	0	222	1	219	6	220
**TP**	0	260	0	242	0	234	0	246	0	218	0	188	0	220	0	219	0	216	0	211
**Sp. Con.**	0	258	0	241	0	233	0	246	0	224	0	193	0	226	0	226	0	222	0	225
**SWT**	0	261	0	242	0	233	0	246	0	225	0	194	0	224	0	224	0	224	0	226
**TN**	0	253	0	235	0	229	0	243	0	218	0	183	0	219	0	216	0	216	1	218
**TSS**	51	251	43	238	40	230	8	244	11	219	5	193	4	224	9	224	5	222	7	225
**Turb**	0	252	0	239	0	230	0	243	0	224	0	194	0	225	0	224	0	224	0	225

Table S2. The monthly mean (x̄), standard deviation (SD), median (Med) and interquartile range (IQR) for dry and wet months of all physicochemical variables analyzed for all stations from November 1999 to October 2019.

Variables		Tributaries								Estuary											
		GORDYRD		C24S49		C23S48		C44S80		SE 09		SE 06		SE 03		SE 02		SE 01		SE 11	
		Dry	Wet	Dry	Wet	Dry	Wet	Dry	Wet	Dry	Wet	Dry	Wet	Dry	Wet	Dry	Wet	Dry	Wet	Dry	Wet
NH_3 mg/L	x̄	0.05	**0.1**	0.04	**0.1**	0.04	**0.1**	0.03	0.07	0.04	**0.09**	0.03	0.07	0.04	**0.09**	0.03	0.08	0.02	0.06	0.01	0.04
	SD	0.05	0.09	0.05	0.1	0.04	0.1	0.03	0.08	0.04	0.07	0.03	0.07	0.04	0.07	0.03	0.08	0.02	0.07	0.02	0.06
	Med	0.05	0.09	0.02	0.08	0.02	**0.09**	0.02	0.04	0.03	**0.07**	0.02	0.05	0.03	**0.07**	0.02	0.06	0.01	0.03	0.005	0.02
	IQR	0.06	0.1	0.05	0.2	0.04	0.2	0.02	0.07	0.05	0.09	0.03	0.09	0.04	0.08	0.03	0.09	0.03	0.08	0.01	0.06
N + N mg/L	x̄	0.09	0.1	0.1	0.05	0.2	0.07	**0.3**	0.2	**0.1**	**0.1**	0.04	0.06	0.08	**0.1**	0.06	0.08	0.04	0.06	0.02	0.04
	SD	0.07	0.1	0.1	0.07	0.2	0.1	0.2	0.2	0.1	0.1	0.05	0.07	0.1	0.1	0.09	0.09	0.07	0.08	0.04	0.06
	Med	0.09	0.09	0.09	0.03	0.1	0.05	**0.3**	0.1	**0.1**	0.07	0.01	0.04	0.03	0.06	0.02	0.05	0.02	0.03	0.006	0.02
	IQR	0.1	0.1	0.2	0.06	0.3	0.08	0.3	0.2	0.2	0.1	0.05	0.09	0.1	0.1	0.09	0.1	0.05	0.09	0.03	0.05
TN mg/L	x̄	0.81	1.2	1.2	**1.4**	1.1	**1.4**	1.2	1.3	1.0	**1.3**	0.78	1.0	0.70	1.1	0.58	0.91	0.46	0.73	0.35	0.55
	SD	0.24	0.41	0.27	0.37	0.34	0.41	0.33	0.38	0.29	0.64	0.18	0.25	0.30	0.34	0.27	0.36	0.29	0.36	0.27	0.36
	Med	0.75	1.2	1.3	**1.5**	1.1	**1.5**	1.2	1.3	0.99	**1.3**	0.80	1.0	0.67	1.0	0.59	0.89	0.47	0.74	0.34	0.52
	IQR	0.34	0.53	0.42	0.56	0.44	0.56	0.40	0.43	0.29	0.30	0.24	0.38	0.39	0.51	0.38	0.50	0.38	0.54	0.27	0.48
OP mg/L	x̄	0.13	**0.28**	0.091	0.23	0.14	0.27	0.062	0.12	0.085	0.14	0.11	**0.20**	0.083	0.16	0.063	0.13	0.038	0.097	0.019	0.052
	SD	0.084	0.22	0.086	0.16	0.099	0.16	0.031	0.086	0.032	0.061	0.046	0.104	0.030	0.075	0.033	0.079	0.033	0.075	0.026	0.055
	Med	0.10	0.22	0.075	0.22	0.11	**0.26**	0.059	0.084	0.081	0.12	0.098	**0.18**	0.078	0.14	0.058	0.12	0.032	0.075	0.013	0.031
	IQR	0.10	0.19	0.097	0.16	0.12	0.24	0.030	0.085	0.037	0.081	0.045	0.13	0.032	0.097	0.026	0.093	0.028	0.087	0.020	0.066
TP mg/L	x̄	0.18	**0.35**	0.14	0.31	0.20	**0.35**	0.13	0.20	0.16	0.21	0.16	**0.27**	0.12	0.22	0.092	0.18	0.061	0.14	0.037	0.084
	SD	0.099	0.23	0.094	0.18	0.11	0.18	0.052	0.098	0.061	0.071	0.053	0.11	0.038	0.089	0.034	0.090	0.037	0.089	0.029	0.072
	Med	0.14	0.31	0.12	0.29	0.17	**0.33**	0.11	0.16	0.15	0.19	0.15	**0.26**	0.12	0.21	0.086	0.16	0.058	0.11	0.032	0.060
	IQR	0.12	0.23	0.12	0.18	0.15	0.27	0.052	0.13	0.052	0.076	0.057	0.14	0.039	0.12	0.030	0.10	0.030	0.10	0.025	0.069
DO mg/L	x̄	4.7	3.0	6.7	4.4	**7.1**	5.3	6.9	5.3	6.6	4.8	6.5	4.6	6.9	5.4	6.9	5.7	6.9	5.8	**7.0**	6.1
	SD	1.8	1.9	1.8	2.7	1.5	1.7	1.5	1.8	1.2	1.7	1.2	1.4	0.91	1.1	0.94	0.99	0.94	0.81	0.95	0.86
	Med	4.6	2.6	6.5	4.0	**7.1**	5.2	6.8	5.0	6.8	4.8	6.5	4.5	3.9	5.3	6.7	5.8	6.9	5.9	**7.0**	6.1
	IQR	2.4	2.2	2.6	3.7	2.0	2.0	2.1	2.5	1.8	2.6	1.7	1.9	1.1	1.2	1.2	1.1	1.0	0.99	0.89	0.83
Color PCU	x̄	38	71	100	**150**	97	149	46	70	51	**103**	44	79	30	69	23	59	14	44	7.8	26
	SD	19	40	60	98	52	90	21	44	31	78	22	39	23	51	23	50	21	45	17	32

	Med	32	62	77	**147**	77	144	39	55	43	68	39	**69**	25	55	16	41	9	26	3	11
	IQR	21	55	83	171	65	174	20	54	23	97	17	55	17	68	16	68	16	60	8	34
TSS mg/L	x̄	3	5	3	4	3	5	12	**13**	10	10	7	8	10	**12**	9	9	9	9	11	8
	SD	7	7	2	5	3	7	16	15	11	8	4	4	8	8	9	6	7	7	9	7
	Med	1	4	2	3	2	4	**8**	**8**	7	7	6	8	8	**11**	6	8	8	7	8	7
	IQR	2	5	3	4	3	4	9	11	6	7	4	5	7	9	6	5	4	5	8	6
Turb. NTU	x̄	2.8	3.8	3.1	3.2	4.4	3.9	**17**	15	**10**	8.8	4.1	5.3	6.7	**10**	4.5	7.1	4.2	6.1	3.9	4.9
	SD	4.2	3.7	1.4	1.8	2.7	2.8	22	17	16	10	1.3	2.0	9.5	9.7	3.2	5.7	2.1	4.5	3.1	4.3
	Med	2.3	2.8	2.9	2.8	3.8	3.3	**9.4**	8.9	4.8	5.6	4.1	5.1	4.2	**7.2**	3.7	5.0	3.7	4.8	3.4	3.9
	IQR	1.8	2.6	1.8	1.1	2.2	1.6	12	9.7	3.5	5.3	1.6	2.2	3.2	5.5	2.2	4.8	2.3	3.1	2.0	3.4
pH**	x̄	7.3	7.1	7.6	7.2	**7.9**	7.2	7.6	7.3	7.6	7.2	7.5	7.3	7.8	7.5	7.9	7.6	**7.9**	7.7	**7.9**	7.8
	SD	0.2	0.2	0.4	0.5	0.3	0.4	0.3	0.4	0.2	0.4	0.3	0.3	0.1	0.3	0.1	0.2	0.2	0.2	0.2	0.2
	Med	7.4	7.2	7.8	7.3	7.8	7.4	**7.8**	7.5	7.6	7.5	7.6	7.4	7.8	7.6	7.9	7.8	**8.0**	7.9	**8.0**	7.9
	IQR	7.6	7.5	7.7	7.1	7.7	7.2	7.9	7.5	7.8	7.6	7.7	7.5	8.1	7.8	8.3	8.1	8.4	8.2	8.3	8.2
Sp. Cond mS/cm	x̄	**2.0**	1.6	1.2	1.1	0.79	0.83	0.74	0.66	11	6.7	15	7.4	31	20	37	27	45	36	**49**	43
	SD	0.51	0.58	0.34	0.45	0.20	0.31	0.25	0.31	8.5	8.5	10	8.8	11	14	11	15	9.0	15	7.1	12
	Med	**2.0**	1.6	1.2	0.97	0.78	0.82	0.70	0.56	11	0.92	13	2.8	32	19	40	28	47	41	**51**	47
	IQR	0.74	0.76	0.47	0.71	0.25	0.41	0.40	0.38	15	12	16	11	13	24	14	26	8.3	23	5.3	16
SWT °C	x̄	20.9	27.8	21.6	28.6	21.7	28.4	22.0	**28.7**	22.1	**28.9**	22.0	28.7	21.6	28.4	21.6	28.3	21.9	27.9	22.3	27.5
	SD	2.9	1.6	2.6	1.7	2.6	1.6	2.7	1.7	2.6	1.7	2.8	2.0	2.7	1.7	2.7	1.6	2.6	1.5	2.4	1.5
	Med	21.1	28.0	21.9	28.8	21.9	28.7	22.0	**29.0**	22.3	**29.0**	22.2	28.8	21.8	28.7	21.8	28.6	22.2	28.2	22.4	27.7
	IQR	4.0	2.0	3.2	2.3	3.4	2.1	3.2	2.1	3.6	2.3	3.7	2.3	3.7	2.3	3.6	2.3	3.4	2.2	2.9	2.0

* Numbers in bold are the largest mean values of a physicochemical variable for a tributary station and an estuary station.

** The average and IQR for pH were determined as the negative logarithm of the average hydrogen ion concentration.
